# Microglial Over-Activation by Social Defeat Stress Contributes to Anxiety- and Depressive-Like Behaviors

**DOI:** 10.3389/fnbeh.2017.00207

**Published:** 2017-10-24

**Authors:** Dirson J. Stein, Mailton F. Vasconcelos, Lucas Albrechet-Souza, Keila M. M. Ceresér, Rosa M. M. de Almeida

**Affiliations:** ^1^Laboratory of Molecular Psychiatry, Hospital de Clínicas de Porto Alegre, Universidade Federal do Rio Grande do Sul, Porto Alegre, Brazil; ^2^Post-Graduate Program in Psychiatry and Behavioral Sciences, Universidade Federal do Rio Grande do Sul, Porto Alegre, Brazil; ^3^Psychology Institute, Universidade Federal do Rio Grande do Sul, Porto Alegre, Brazil

**Keywords:** microglia, neuroimmunity, immune cells, psychosocial stress, neuropsychiatric disorders, inflammatory processes

## Abstract

Hyper activation of the neuroimmune system is strongly related to the development of neuropsychiatric disorders. Psychosocial stress has been postulated to play an important role in triggering anxiety and major depression. In preclinical models, there is mounting evidence that social defeat stress activates microglial cells in the central nervous system. This type of stress could be one of the major factors in the development of these psychopathologies. Here, we reviewed the most recent literature on social defeat and the associated immunological reactions. We focused our attention on microglial cells and kept the effect of social defeat over microglia separate from the effect of this stressor on other immune cells and the influence of peripheral immune components in priming central immune reactions. Furthermore, we considered how social defeat stress affects microglial cells and the consequent development of anxiety- and depressive-like states in preclinical studies. We highlighted evidence for the negative impact of the over-activation of the neuroimmune system, especially by the overproduction of pro-inflammatory mediators and cytotoxins. Overproduction of these molecules may cause cellular damage and loss or decreased function of neuronal activity by excessively pruning synaptic connections that ultimately contribute to the development of anxiety- and depressive-like states.

## Introduction

Neuropsychiatric disorders, such as anxiety and major depression (MD), are highly prevalent and contribute significantly to the worldwide burden of diseases (Ferrari et al., 2013; Whiteford et al., 2013). As a major contributor to the development of affective and neuropsychiatric disorders in humans, psychosocial stress has been reported to induce central and peripheral immune pathway signaling by repeated activation of the neuroendocrine and neurovegetative systems (Glaser and Kiecolt-Glaser, 2005; Lehmann et al., 2016). When the individual is repeatedly exposed to stress, the brain homeostatic environment alters and may give rise to various cognitive and mood disorders that impair everyday functioning and overall quality of life (McKim et al., 2016a). Within the central nervous system (CNS) immunological defense, microglia are the key immune players and acquire a reactive profile to cope with altered homeostasis (Hanisch and Kettenmann, 2007). When activated, these cells are supposed to trigger anxiety- and depressive-like behaviors (Lehmann et al., 2016), mainly by increasing the expression of pro-inflammatory mediators and neurotoxins in stress-sensitive brain regions (Reader et al., 2015; Ramirez and Sheridan, 2016), and can ultimately influence the overall cellular functions and survival, from neurons to glial cells.

Brief and prolonged episodes of social defeat (SD) have been correlated with anxiety- and depressive-like behaviors, respectively. While brief episodes can increase self-grooming, locomotion in novel environments, risk assessment and binge-like cocaine self-administration, prolonged episodes induce anhedonic behaviors such as reduced sweet solution preference, reduced mounting in copulatory behavior, reduced climbing in the forced swimming test (FST), lower general activity and sociability and suppressed cocaine intake (Razzoli et al., 2009; Miczek et al., 2011; Hollis and Kabbaj, 2014; Vasconcelos et al., 2015). Despite the clear evidence of the role of social stress triggering mood disorder-related behaviors, to the best of our knowledge, the exclusive contribution of SD to microglial over-activation has never been reviewed. Here, we discuss the emerging field of social stress-induced microglial over-activation, providing an overview of how microglial reactions can lead to these mood disorders, and briefly discuss some relevant translational significance of the findings. We hypothesized that acute/repeated and chronic social defeat (CSD) stress can induce microglial activation and over-activation that can engender anxiety and depressive-like states, respectively. The repeated social defeat (RSD) paradigm reported in this review is characterized by the introduction of an aggressive intruder male into the cages of established male cohorts of mice for three or six consecutive nights, leading to the establishment of dominance over the original colony (Wohleb et al., 2014b). CSD varied from 14 to 20 days of a 24 h/day dyadic social housing, exposing the defeated animal to continuous psychological stress via sensory interaction with the aggressor, accompanied by a 5 min/day agonistic encounter between the aggressor and the defeated animal (Brachman et al., 2015; Lehmann et al., 2016; Tong et al., 2017).

Articles used in this mini-review were selected from the PubMed, Embase and ScienceDirect databases between March and April 2017. Search terms were “microglia” and “SD”, without any time limitation. Of the 23 selected articles, 11 were excluded for the following reasons: not an original article, no clear effect of stress over microglia and the use of mixed stress protocols.

## Microglia: The First Defence of The CNS

Microglia comprise about 10%–15% of all brain cells and are crucial players in normal development through the regulation of functional and structural processes, contributing to plasticity from individual synapses to neural circuits and behavior (Wake et al., 2013; Salter and Beggs, 2014; Verkhratsky et al., 2015). Microglial cells originate from extra-embryonic yolk sac progenitors, establish unique CNS cell populations and are maintained throughout life by local proliferation (Ginhoux et al., 2010, 2013). As tissue-resident macrophages in the CNS, along with other mononuclear phagocytes, microglia are critical effectors and regulators of changes in CNS homeostasis during development, in health and disease (Hanisch and Kettenmann, 2007; Prinz and Priller, 2014).

Some evidence points to new and fundamental roles for microglia in the control of neuronal proliferation and differentiation, as well as in the formation of synaptic connections (Kettenmann et al., 2011; Ginhoux et al., 2013). These cells are distributed in the brain parenchyma, have small delineated processes and actively screen the inter-neuronal space for incoming threats, exhibiting immune regulatory functions, from local surveillance to the removal of debris (Prinz and Priller, 2014). Microglial activation is the main neuroinflammatory element in the CNS, providing the front line defense whenever injury, disease or infection occurs (Lehnardt, 2010; Tang and Le, 2016).

Inflammatory processes are usually self-limited, culminating with tissue repair; damage to the CNS occurs when the system is over-activated for a long time, extending the release of pro-inflammatory mediators and neurotoxins. This process can worsen tissue damage and negatively impact disease outcome, leading to anxiety- and depressive-like states (Reader et al., 2015; Ramirez and Sheridan, 2016). Increasing evidence points to a heterogeneous status of microglial activation in the CNS. Although it is not a consensus, some authors categorize microglia into two opposite activation states, M1 and M2 phenotypes, which can produce either cytotoxic or neuroprotective effects (Tang and Le, 2016). M1-polarized microglia are associated with the production of pro-inflammatory cytokines such as tumor necrosis factor-α (TNF-α), interleukin-1β (IL-1β), interleukin-6 (IL-6), superoxide, nitric oxide, reactive oxygen species and proteases (Ajmone-Cat et al., 2013), whereas M2-polarized microglia express cytokines and receptors that are implicated in the inhibition of inflammation and restoration of homeostasis by tissue repair and extracellular matrix reconstruction (Nakagawa and Chiba, 2014; Tang and Le, 2016). Nevertheless, as this nomenclature is not fully accepted and some authors consider microglia polarization to have derived from studying peripheral macrophages rather than microglia (Ransohoff, 2016), it is important to carefully use and interpret these terms to avoid misunderstandings.

## The SD Paradigm as a Valid Stressor

Most stressors in human life arise from interactions within the social environment. In fact, social stress encompasses various types of significant life events, ranging from maternal separation (Meaney, 2001; Nishi et al., 2014), brief episodes of social confrontations in adolescence and adulthood, to continuous subordination stress (Miczek et al., 2008). In preclinical studies, some models of stress are often criticized as being artificial and not representative of human stress (Björkqvist, 2001; Almeida et al., 2002).

The SD paradigm is recognized as an ethological valid method to engender social stress in rodents (Vasconcelos et al., 2015; Henriques-Alves and Queiroz, 2016; Koolhaas et al., 2017). RSD is a stressor that recapitulates key physiological, immunological and behavioral alterations observed in humans exposed to chronic psychosocial stress (McKim et al., 2016a). Models of psychosocial stress rely on innate social behavior among pairs or groups of male rodents allowing the formation of stable dominant-subordinate relationships (Krishnan and Nestler, 2011). Another strong point of these models is the lack of habituation; despite repeated exposures, animals continue to generate emotional stress responses (Tidey and Miczek, 1997).

SD stress activates the hypothalamic-pituitary-adrenal axis and sympathetic nervous system, increasing systemic glucocorticoids that trigger the release of catecholamines and pro-inflammatory cytokines (Avitsur et al., 2001; Herman et al., 2016). Although there are distinct models of social stress, this review will focus on the role of SD in the development of anxiety and MD, tracking the contribution of the over-activation of the main CNS immune component, microglia, in triggering these psychiatric diseases.

## Effects of SD Stress on Microglial Cells

One of the major advances in the field of the study of psychiatric disorders came from the notion that the immune system and inflammatory processes can be activated by psychosocial stressors (Miller and Raison, 2015). Despite the well-established evidence that the peripheral and central immune systems act in concert to promote the stress reaction, greater attention has been given to immune cells of the CNS, in particular, microglia. Social stress may activate microglial cells in a way different from other stressors (Glaser and Kiecolt-Glaser, 2005; Calcia et al., 2016) and seems to exert a direct effect over microglia activity through the activation of glucocorticoid and mineralocorticoid (Sierra et al., 2008) and β-adrenergic receptors (Walker et al., 2013; Calcia et al., 2016). Considering these factors, we directed our attention to microglial reactions induced by SD; the evidence is presented in Table 1.

**Table 1:** Microglial activation profile induced by SD stress and related behavioral outcomes.

Defeated subjects	Age	Stressor	Source of microglia	Microglia changes	Behavioral outcomes	Reference
♂ICR mice	8–10 w	CSD (20 days)	HPC (DG) mPFC	↓ hippocampal microglial numbers, process lengths and soma areas No changes in microglia numbers in PFC ↓ expression levels of Iba-1 and CD11b	↑ immobility in the TST and FST ↓ in sucrose consumption in the SPT ↓ time in the center in the OFT	Tong et al. (2017)
♂C57BL/6J mice ♂CX_3_CR_1_^wt/gfp^ mice	8–10 w	Acute RSD (3 days) Chronic RSD (14 days)	Infralimbic ctx Prelimbic ctx Anterior cingulate ctx Piriform ctx Nucleus accu. Dorsal DG Basolateral AMY	Chronic SD microglia phagocytosed more labeled material ↑ numbers of CD68^hi^ microglia in chronic SD mice *vs* HC and ASD ↑ microglial phagocytosis *ex vivo* ↑ microglial proliferation after ASD	↓ marking preference in the USM task in CSD CX_3_CR_1_^wt/gfp^ mice ↓ SI in CSD mice	Lehmann et al. (2016)
♂C57BL/6 mice	6–8 w	RSD (6 days)	HPC (DG)	↑ soma size, shorter and thicker cell processes ↑ microglial Iba-1 immunoreactivity, augmented in caudal HPC ↑ IL-1β mRNA	↑ latency and distance to reach the platform of the MWM ↑ time in the outer annulus of the MWM ↓ time in the target quadrant of the MWM ↑ latency to find the escape hole in the BM ↑ number of errors to reach the escape hole in the BM	McKim et al. (2016a)
♂C57BL/6 mice	6–8 w	RSD (6 days)	Whole brain homogenates	↑ microglia gene expression of IL-1β, IL-6, TNF-α ↑ microglia activation markers TLR-4, CCL_2_ and CX_3_CR_1_	↓ number of center entries in the OFT ↑ time to first enter the center in the OFT ↓ time spent in the interaction zone in the SAT	Ramirez and Sheridan (2016)
♂C57BL/6 mice	6–8 w	RSD (6 days)	HPC HPT Whole brain homogenates	↑ microglia gene expression and mRNA expression of IL-1β, IL-6, TNF-α ↑ microglia activation markers TLR-4, CCL_2_ and CX_3_CR_1_	↓ number of center entries in the OFT ↑ time to first enter the center in the OFT ↓ time spent in the interaction zone in the SAT	Ramirez et al. (2016)
Donors: ♂C57BL/6 mice Recipients: ♂Rag2^−/−^ mice	10–13 w	CSD–(14 days)	Whole brain homogenates (-cerebellum)	Stressed/lymphocytes donors: ↑ basal gene expression of the M1 microglia cytokines IL-1β and IL-6. ↓ basal expression of the M2 marker MRC1 and muted ARG response to IL-4 Non-stressed/lymphocytes recipients: skewed microglia to a M2-like phenotype. ↑ ARG expression (basal and IL-4 stimulated) and a generally muted response of IL-1β and IL-6 to LPS stimulation	SD → Rag mice on C57BL/6 background: ↑ transitions in the L/D box ↑ center time in the OFT ↑ interaction in the SI test ↑ time mobile in the TST SD → Rag mice on 129 background: ↑ transitions in the L/D box ↑ travel in the OFT ↑ center time in the OFT ↑ marking preference in the USM	Brachman et al. (2015)
♂C57BL/6 mice	6–8 w	RSD (6 days)	Whole brain homogenates	↑ microglia gene expression of IL-6 ↑ gene expression of IL-1β, IL-6, and TNF-α in *ex vivo* LPS stimulated microglia from RSD mice ↑ relative gene expression of IL-6 in microglia 24 days after stress cessation	↓ time spent in the interaction zone in the SAT ↑ time spent in the corners in the SAT	Ramirez et al. (2015)
♂C57BL/6 WT mice ♂IL-1R1^Ko^ mice ♂IL-1R1^Kd^ mice	6–10 w	RSD (6 days)	Whole brain homogenates	Robust change in the morphology of microglia in WT mice after RSD ↑ surface expression of the activation marker CD14 Altered gene expression of inflammatory-related genes in brain CD11b^+^ cells De-ramified morphology in WT and IL-1R1^Kd^ mice with increased Iba-1 proportional area	WT: ↑ thigmotaxis in the OFT ↑ latency to enter the center in the OFT ↓ time spent in the center in the OFT ↓ time to enter the dark zone in the L/D box ↑ time spent in the dark zone of the L/D box	Wohleb et al. (2014b)
♂C57BL/6 WT mice	6–10 w	ASD (1 day) RSD (6 days)	PFC AMY HPC (CA3 and DG) Whole brain homogenates	ASD altered microglia morphology in stress sensitized mice ↑ Iba-1 proportional area in PFC, AMY and HPC (CA3 and DG) in RSD mice ↑ mRNA levels of IL-1β, TNF-α and CD14, 5 days after RSD ↓ CX_3_CR_1_ after RSD	↑ latency to enter the center in the OFT at 0.5 and 8 days ↓ time spent in the center in the OFT at 0.5 days ↓ time spent in the interaction zone in the SAT at 0.5, 8 and 24 days ↑ time spent in the corners in the SAT at 0.5 and 24 days ↓ % time spent in the interaction zone in the SAT	Wohleb et al. (2014a)
♂C57BL/6 mice	6–10 w	SSD (1 day) RSD (3 or 6 days)	PFC HPC (DG)	↑ Iba-1 immunoreactivity after RSD ↑ microglial activation (de-ramified morphology) in PFC and DG after RSD Microglia recruit peripheral myeloid cells to the brain	↑ latency to enter the center in the OFT after RSD (3 and 6 days) ↓ time spent in the center in the OFT after RSD (3 and 6 days) ↓ time to enter the dark zone in the L/D box after RSD (6 days) ↓ time spent in the light zone in the L/D box after RSD (6 days)	Wohleb et al. (2013)
♂C57BL/6 mice	6 w	RSD (6 days)	PFC HPC PVN AMY Whole brain homogenates	↑ mRNA levels of IL-1β, TNF-α, iNOS and CD14 ↑ IL-1β and TNF-α mRNA levels after LPS injection Hypertrophic microglia with shorter and thicker processes ↑ inflammatory response of brain CD11b^+^ cells following a peripheral LPS challenge ↑ activated morphology of microglia (Iba-1^+^) in the PFC, AMY, PVN, and HPC Amplified surface expression of CD14 after LPS injection	↓ social exploratory behavior ↓ time spent in the center in the OFT ↑ latency to enter the center in the OFT	Wohleb et al. (2012)
♂C57BL/6 mice ♂IL-1R1^−/−^ mice	6–8 w	SSD (1 day) RSD (3 days) RSD (6 days)	AMY PFC HPC	↑ de-ramified (shorter and thicker processes by Iba-1) microglia in MeAMY, PFC and HPC ↑ surface markers CD14, CD68 and TLR-4 after RSD (6d) ↑ mRNA expression of interleukin IL-1β ↓ levels of glucocorticoid responsive genes (GILZ and FKBP51) ↑ *ex vivo* levels of IL-6, TNF-α and MCP-1 following LPS stimulation	WT mice: ↓ time to enter the dark zone in the L/D box after RSD (6 days) ↑ time spent in the dark zone in the L/D box after RSD (6 days)	Wohleb et al. (2011)

*Abbreviations: ASD, Acute Social Defeat; AMY, Amygdala; BM, Barnes Maze; CCL_2_, Chemokine (C-C motif) ligand 2; CSD, Chronic Social Defeat; Ctx, cortex; CXCL_2_, Chemokine (C-X-C motif) ligand 2; d, days; DG, Dentate Gyrus; FKBP51, FK506 binding protein 51; FST, Forced Swimming Test; GILZ, Glucocorticoid-induced leucine zipper; HPC, Hippocampus; Iba-1, Ionized Calcium-Binding Adapter Molecule 1; IL-1R1^Kd^, IL-1 receptor type-1 knock-down; IL-1R1^Ko^, IL-1 receptor type-1 knock-out; L/D, Light/Dark; LPS, Lipopolysaccharide; MCP-1, monocyte chemoattractant protein-1; mPFC, medial Pre-Frontal Cortex; MWM, Morris Water Maze; OFT, Open-Field Test; PFC, Pre-Frontal Cortex; PND, Post-Natal Day; PVN, Paraventricular Nucleus; RSD, Repeated Social Defeat; SAT, Social Avoidance Test; SI, Social Interaction; SSD, Single Social Defeat; Tg, Transgenic; TST, Tail Suspension Test; USM, Urine Scent Marking; w, weeks; WT, Wild-Type*.

**Figure 1:**
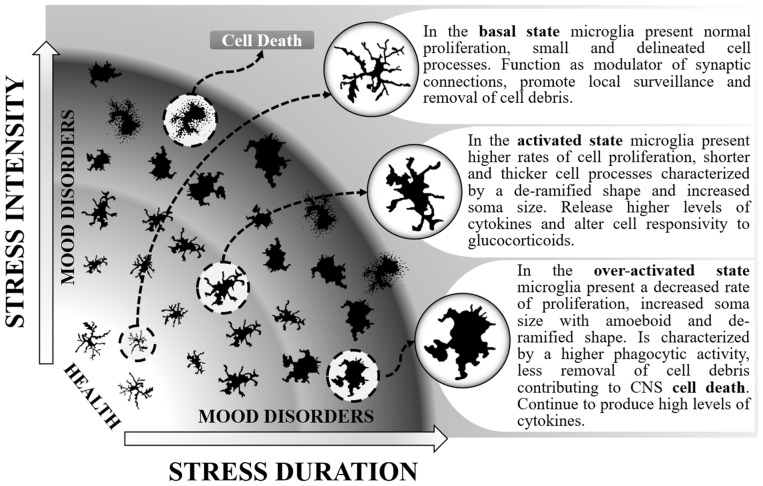
Different stages of microglial activation caused by changes in the intensity and duration of social stressors that can maintain individuals in a healthy state or contribute to both anxiety and depressive-like behaviors. In the basal state, microglia can be distinguished by normal levels of immunoreactivity to ionized calcium-binding adapter molecule 1 (Iba-1) and CD11b. In this state, enable proper coping to stress situations. When activated, microglia proliferate, release higher levels of interleukin-1β (IL-1β), interleukin-6 (IL-6) and tumor necrosis factor-α (TNF-α), present higher expression of toll-like receptor 4 (TLR-4), chemokine (C-C motif) ligand 2 (CCL_2_), CX_3_CR_1_) and decreased levels of glucocorticoid-induced leucine zipper (GILZ) and FK506 binding protein 51 (FKBP51). They can be distinguished by higher immunoreactivity to Iba-1, CD11b, CD14 and CD68. During the activated state, the release of pro-inflammatory mediators and the altered response to glucocorticoids may lead to anxiety. The activated state can also be protective by resuming stress effects. As a consequence, microglia return to their basal state. Otherwise, persistent stress shifts microglia to an over-activated state. Overactive microglia continue to release pro-inflammatory mediators (IL-1β and IL-6). They can be distinguished by higher immunoreactivity to CD68^hi^ and possibly lower levels of CX_3_CR_1_ and iNOS antibodies. Along with the microglial phagocytic activity occurs a higher rate of cell death, including microglia as well as neuronal and other glial cells. This effect reduces their capacity to remove cell debris and surveillance of the inter-neuronal space, altogether leading to depression.

Microglia present increased activation status after SD (Wohleb et al., 2014b; Ramirez and Sheridan, 2016) and the effects are mainly observed within brain regions associated with fear, anxiety and threat appraisal (Wohleb et al., 2015). From a ramified aspect found in the immunosurveillant state, microglia change robustly to a de-ramified state with shorter and thicker processes (Wohleb et al., 2011, 2012, 2013, 2014b), leading to increased soma size after acute, RSD and CSD (McKim et al., 2016a; Figure 1). Changes in soma and processes are usually analyzed by increases in ionised calcium-binding adapter molecule 1 (Iba-1) or cluster of differentiation 11b (CD11b) immunoreactivity. However, although the vast majority of studies report results similar to those described above, decreases in microglial Iba-1, CD11b and consequently soma areas were found by others in the dentate gyrus (DG), but not in the medial prefrontal cortex, in a stress protocol that consisted of 20 days of exposure to SD (Tong et al., 2017). These controversial data could be attributed to differences in stress chronicity.

One additional way to identify changes in microglia activity is through the analysis of activation markers such as the chemokine (C-C motif) ligand 2 (CCL_2_), toll-like receptor 4 (TLR-4) or the CX_3_ chemokine receptor 1 (CX_3_CR_1_) which are expressed by microglial cells. SD induces an increase in the gene expression of TLR-4, CCL_2_ and CX_3_CR_1_ (Ramirez et al., 2015, 2016; Ramirez and Sheridan, 2016). However, decreases in CX_3_CR_1_ gene expression were also observed after SD, although in enriched brain CD11b^+^ cells (Wohleb et al., 2014a). One of the most evident reactions to SD observed in microglial cells is the rise in gene expression and mRNA levels of the pro-inflammatory cytokines IL-1β, IL-6 and TNF-α and expression of the surface activation marker CD14. Increases of these inflammatory mediators were observed after acute, RSD and CSD (Wohleb et al., 2011, 2012, 2014a; Brachman et al., 2015; Ramirez et al., 2015, 2016; McKim et al., 2016a; Ramirez and Sheridan, 2016), even 24 days after stress cessation (Ramirez et al., 2015). The importance of these findings is reinforced by the results obtained from either microglial cells analyzed in fresh CNS tissue, isolated from socially defeated animals (Wohleb et al., 2012) or in *ex vivo* SD-sensitized microglia stimulated with lipopolysaccharide (LPS; Wohleb et al., 2011). Additionally, reduced levels of glucocorticoid responsive genes (GILZ and FKBP51) are evident after exposure to SD (Wohleb et al., 2011). Chronically SD stress-activated microglial cells increase their phagocytic activity. This effect is achieved by increasing the expression of CD68^hi^ (a marker for phagocytic activity; Lehmann et al., 2016). The increasing phagocytic activity of microglia from CSD animals suggests that cellular debris or cell damage or death may be a hallmark of chronic stress effects on the brain. SD can also change microglial cell numbers; while acute SD enhances the number of microglia (Lehmann et al., 2016), CSD diminishes these cells (Tong et al., 2017), mainly in the hippocampus. It seems that a crucial factor is the intensity of activation of microglia by stress, which can lead to different psychiatric disorder outcomes (Figure 1). Taken together, these data highlight the broad spectrum of effects that can be observed in microglial cells when activated by SD.

## The Link Between Microglial Activation, Anxiety- and Depressive-Like Behaviors

It is now well known that disturbances in microglial functioning has an etiological role in mood disorders (Frick et al., 2013; Kreisel et al., 2014). However, if the effect of social stress on these deregulated behaviors can be mainly attributed to microglial over-activation or if the participation of other CNS immune cells and/or the peripheral immune system plays a major role remains controversial. While researchers have shown in some studies that SD stress-induced anxiety- and depressive-like states are mediated by the activation of microglia with the involvement of peripheral macrophages and trafficking of monocytes to the brain (Wohleb et al., 2013, 2014b, 2015), other studies excluded the direct involvement of peripheral monocytes triggering these behaviors (Lehmann et al., 2016). Stress chronicity and/or peripheral wounds (triggers of peripheral immune reactions), which can usually be observed in defeated animals after confrontation with an aggressor, could be major determinants. This is one of the main reasons that led researchers to choose alternative stress protocols, such as variable unpredictable stress and foot shocks to study microglial activation in neuropsychiatric disorders, even though these procedures present lower ethological relevance.

Studies in humans have shown that microglial activation is positively correlated with psychiatric disorders. For example, individuals experiencing a major depressive episode present enhanced positron emission topography labeling of the translocator protein (TSPO), a putative marker of neuroinflammation and microglia activation (Setiawan et al., 2015). It has also been speculated that there is a causal link between microglial activation and suicidal behavior (Schnieder et al., 2014); neuroendocrine factors, cytokines and nitric oxide, which are released from microglial cells and are known to modulate noradrenergic or serotonergic neurotransmission, may trigger suicidal behavior (Steiner et al., 2008). Pro-inflammatory cytokines including IL-1β and TNF-α, can reduce the availability of serotonin, dopamine and noradrenaline by increasing the expression and function of reuptake transporters, reducing synthesis or decreasing monoamine precursors (Miller and Raison, 2015). Activated microglia can also act on the glutamate pathway and together with astrocytes stimulate the increased release of this neurotransmitter and decreased brain-derived neurotrophic factor, which ultimately leads to excitotoxicity (Steiner et al., 2012; Miller and Raison, 2015). Additionally, it has been shown that elevated pro-inflammatory cytokine levels caused by microglia activation associated with the recruitment of monocytes to the brain contribute to the development and persistent anxiety-like behavior (Wohleb et al., 2014b, 2015). Moreover, chronic microglial activation in particular can result in neuronal apoptosis, neurogenesis inhibition, hippocampal volume reduction, lower neurotransmitters synthesis and cytotoxicity (Ascoli et al., 2016), which is ultimately related to depressive behavior.

Although microglia are not the only effectors of the immune system, it has been suggested that the anti-inflammatory effect of antidepressants may have protective effects by silencing RSD-induced priming and activation of microglia, thus down-regulating the biosynthesis of high levels of pro-inflammatory cytokines (Ramirez et al., 2015). Recently, microglia have been recognized as important targets for pharmaceutical research. Brain diseases, including depression and anxiety, could potentially be treated with drugs that are capable of inhibiting or restoring specific microglial functions (Biber et al., 2016). Anti-inflammatory drugs such as COX2 inhibitors or minocycline, aimed at inhibiting the pro-inflammatory status of microglia, have been suggested as therapeutics for inflammatory brain diseases (Biber et al., 2016). The CX_3_CR_1_, as an exclusive microglial marker, could also be a potential target. Since the activation of microglia is not consistent for all patients, it has been recently proposed that anti-inflammatory treatment targeting microglial activation could specifically be more effective in patients with increased microglial activation, leading to the idea that microglial activation may be a marker for severe and untreatable psychiatric disorders (Mondelli et al., 2017).

Social stress can alter the number of microglial cells (Lehmann et al., 2016; Tong et al., 2017), mainly dependent on the duration of stress exposure. While acute, but not CSD is supposed to increase microglial proliferation selectively in telencephalic stress-related brain areas (Lehmann et al., 2016), a loss of hippocampal microglia was observed and is supposed to promote the development of MD, indicating that the restoration of microglial functions and/or numbers may be beneficial for the therapy of MD (Tong et al., 2017). Since pro-inflammatory cytokines can also modify neurogenesis in the hippocampus (Koo and Duman, 2009), RSD has been shown to induce anxiety-like behavior by impairing the neuronal differentiation of neural progenitor cells in the hippocampus that proliferated during stress exposure. These data were positively correlated to an impairment in performance on working and spatial memory in the Morris water maze (MWM) and transiently disrupted short-term memory recall in the Barnes maze (BM; McKim et al., 2016a). Overall, these data highlight the magnitude of the microglial over-activation-induced deficits in monoamine neurotransmission, cytotoxicity, cellular loss and reduced neurogenesis, ultimately leading to memory impairment and behaviors that are observed in both, anxiety and depression.

## Conclusion Remarks

Exposure to SD induces microglial cells to assume an activated state, which initially may be considered beneficial. RSD and CSD can induce microglia to assume over-activated states that, by persistently releasing pro-inflammatory mediators, cytotoxins and reactive oxygen species, may cause cellular dystrophy and a loss or decreased function of neuronal activity through excessively pruned synaptic connections. All of these stress effects over microglia worsen memory and behaviors that are important factors in psychiatric disorders. The SD paradigm is an important tool to induce anxiety- and depressive-like states in laboratory animals for investigating stress-induced immunological and behavioral alterations.

It seems that the development of anxiety and MD is, besides microglial activation, dependent on peripheral monocyte recruitment to the brain (McKim et al., 2016b), attaching importance to the bidirectional communication between the brain and peripheral immune system. However, since the activation of microglia by psychosocial stress might be different from that of physical injury (Glaser and Kiecolt-Glaser, 2005), more attention must be given to peripheral wounds when studying SD stress effects over central immune reactions. SD protocols that allow physical injuries to the defeated animal during confrontations with an opponent may contribute to the participation of peripheral immune cells in the final outcome. Alternatively, stress protocols that do not involve physical injuries, such as chronic unpredictable stress, can be used to overcome this issue. Contradictory findings have shown that microglial over-activation, as well as microglial dystrophy and loss, can mediate the development of MD. Depression is considered to be a disorder that is associated with microglial over-activation. That leads to an interpretation that suppressed microglial hyperactivity should be the focus to treat depressive symptoms (Tong et al., 2017). However, since microglia in its basal state is also critical for brain normal function, microglial dystrophy and loss would also mediate the development of this disorder (Kreisel et al., 2014; Tong et al., 2017). Therefore, over-inhibition or over-down-regulation of microglial function will inevitably produce detrimental effects as well. Focusing on microglial cells as therapeutic targets for pharmacological interventions, especially by restoring functions and/or basal levels, may be a promising strategy for anxiety and depression therapy.

## Author Contributions

All authors contributed equally to this study. All of them contributed to the conception and design of the work, literature analyses and interpretation, drafting the article, critical revision and final approval of the version to be published.

## Conflict of Interest Statement

The authors declare that the research was conducted in the absence of any commercial or financial relationships that could be construed as a potential conflict of interest.
